# CT-derived quantitative imaging biomarkers for hypoxemia risk in repeated lung resection: a retrospective study

**DOI:** 10.1186/s12880-026-02528-4

**Published:** 2026-06-27

**Authors:** Jing Peng, Jinde Wang, Xingxiang Dong, Jinhu Miao, Han Yang, Chulin Wang, Gang Guo, Ying Wang, Yingxin Yang, Qisheng Wei, Siyu Xiong, Li Zhao

**Affiliations:** 1 Department of Anesthesiology, The Third Affiliated Hospital of Kunming Medical University, Yunnan Cancer Hospital, Peking University Cancer Hospital Yunnan, No. 519 Kunzhou Road, Xishan District, Kunming, Yunnan Province 650118 China; 2 Department of Radiology, The Third Affiliated Hospital of Kunming Medical University, Yunnan Cancer Hospital, Peking University Cancer Hospital Yunnan, No. 519 Kunzhou Road, Xishan District, Kunming, Yunnan Province 650118 China; 3 Department of Operation Room, The Third Affiliated Hospital of Kunming Medical University, Yunnan Cancer Hospital, Peking University Cancer Hospital Yunnan, No. 519 Kunzhou Road, Xishan District, Kunming, Yunnan Province 650118 China; 4 Department of Thoracic Surgery II, The Third Affiliated Hospital of Kunming Medical University, Yunnan Cancer Hospital, Peking University Cancer Hospital Yunnan, No. 519 Kunzhou Road, Xishan District, Kunming, Yunnan Province 650118 China; 5 Department of Clinical Medicine, Kunming Medical University, Kunming, 650500 China

**Keywords:** Quantitative imaging biomarker, CT-derived functional lung volume, hypoxemia, One-lung ventilation, Repeated lung resection, Preoperative risk stratification

## Abstract

**Background:**

This study sought to elucidate CT-derived quantitative imaging biomarkers and clinical parameters predisposing to arterial oxygen desaturation during one-lung ventilation (OLV) in individuals undergoing repeated lung resections, to improve understanding of preoperative factors associated with hypoxemia risk during OLV in this population.

**Methods:**

A retrospective analysis was performed on 139 patients who underwent repeated lung resections between January 2022 and December 2023 at Yunnan Cancer Hospital. Preoperative CT-derived quantitative imaging biomarkers, including functional lung volume percentage (FLV%) and low attenuation volume percentage (LAV%), were extracted from routine CT scans alongside other clinical variables. Thirteen potential risk factors were first examined using Pearson’s correlation and univariate regression analysis to identify variables significantly associated with hypoxemia. Variance inflation factor (VIF) tests were applied to exclude multicollinearity before performing multivariable logistic regression to determine independent risk factors.

**Results:**

The incidence of hypoxemia during OLV in repeated lung resections was 16.55%. Four independent risk factors were identified: proportion of functional lung volume in the dependent lung as a key quantitative imaging biomarker (FLV%-dependent lung: OR 0.92, 95% CI 0.85–0.99; *p* = 0.038), a history of prior hypoxemia (OR 7.64, 95% CI 1.65–35.31; *p* = 0.009), moderate to severe postoperative pain at 48 hours (OR 5.59, 95% CI 1.95–16.03; *p* = 0.001), and forced expiratory volume in one second (FEV1% predicted: OR 1.04, 95% CI 1.00–1.08; *p* = 0.038).

**Conclusion:**

CT-derived quantitative imaging biomarker FLV%-dependent lung and FEV_1_% predicted, a history of prior hypoxemia and moderate-to-severe acute postoperative pain within 48 hours after the initial lung resection, are associated with hypoxemia risk in repeated lung resections.

## Background

Among all malignancies, lung cancer ranks first in terms of worldwide incidence and mortality [1]. The increased availability of chest CT scans and enhanced lung cancer survival rates have resulted in a rise in the incidence of multiple primary lung cancers (MPLC), making repeated lung resection an increasingly common therapeutic strategy for patients without extra-thoracic metastases and with adequate residual lung function [2]. Notably, multiple resections account for 20–30% of MPLC cases in Asian non-smoking populations [3]. However, this expanding surgical population presents distinct physiological challenges: unlike primary resection, where hypoxemia during one-lung ventilation (OLV) mainly arises from ventilation/perfusion mismatch and intrapulmonary shunting [4–6], repeated resection is further compounded by prior parenchymal loss and compensatory redistribution of blood flow and ventilation [7]. These altered mechanics render patients undergoing repeated lung resection particularly vulnerable to OLV-related hypoxemia, which independently predicts increased postoperative complications and mortality [8]. Consequently, identifying preoperative risk factors specific to this understudied, high-risk cohort has become a critical priority for anesthesiologists.

Several studies have identified risk factors of hypoxemia during OLV, including preoperative factors (history of lung resection, DLCO, age, hematocrit) [9–11], intraoperative physiological parameters (oxygen reserve index, ventilation efficiency, PETCO_2_ differential) [7, 12, 13], and perfusion-related variables [10]. However, these studies predominantly relied on conventional clinical or intraoperative measurements, with limited exploration of quantitative imaging biomarkers. Moreover, which preoperative factors—particularly CT-derived biomarkers—predict hypoxemia specifically in repeated lung resection remains unknown. This knowledge is crucial for guiding anesthesiologists in selecting appropriate lung isolation tools (double-lumen tube [DLT] or bronchial blocker) for patients undergoing repeated lung resection [14–16].

The advent of CT technology has enabled digital quantification of lung tissue, facilitating extraction of quantitative imaging biomarkers from routine preoperative scans. Quantitative CT assessment leverages functional lung volume (FLV) and low attenuating volume (LAV) to mirror ventilatory efficiency, peripheral perfusion, and emphysema severity [17, 18]. FLV permits preoperative assessment of localized lung function (e.g., unilateral lung/single lobe)—directly relevant to OLV, which isolates ventilation to the dependent lung—unlike pulmonary function tests (PFT) that only provide global measurements [19]. Fan et al. [20] defined FLV% as the percentage of FLV in the proposed resected lobe relative to total lung FLV, thereby quantifying the functional contribution of resected tissue. These biomarkers offer objective; reproducible measures of regional lung function that conventional assessments cannot provide [21]. Growing evidence confirms that FLV% and LAV% can assess functional status under different diseases or after lung surgery [7, 22–26]. Our previous study demonstrated that FLV% and LAV% measured prior to initial lung cancer resection were independent risk factors of subsequent OLV-related hypoxemia [27]. However, repeated lung resection presents distinct physiological challenges: prior parenchymal loss and compensatory redistribution of ventilation and perfusion alter the functional anatomy, potentially affecting the predictive validity of these biomarkers. Consequently, whether FLV% and LAV% retain their predictive value in this altered anatomical context remains unknown.

To our knowledge, studies specifically exploring preoperative CT-derived quantitative imaging biomarkers in patients undergoing repeated lung resection remain scarce. We sought to explore whether preoperative FLV% and/or LAV% are independently associated with hypoxemia during OLV in this high-risk population. This study addresses this gap through an exploratory analysis investigating these preoperative biomarkers alongside clinical variables to identify potential risk factors for hypoxemia.

## Methods

**Fig. 1:**
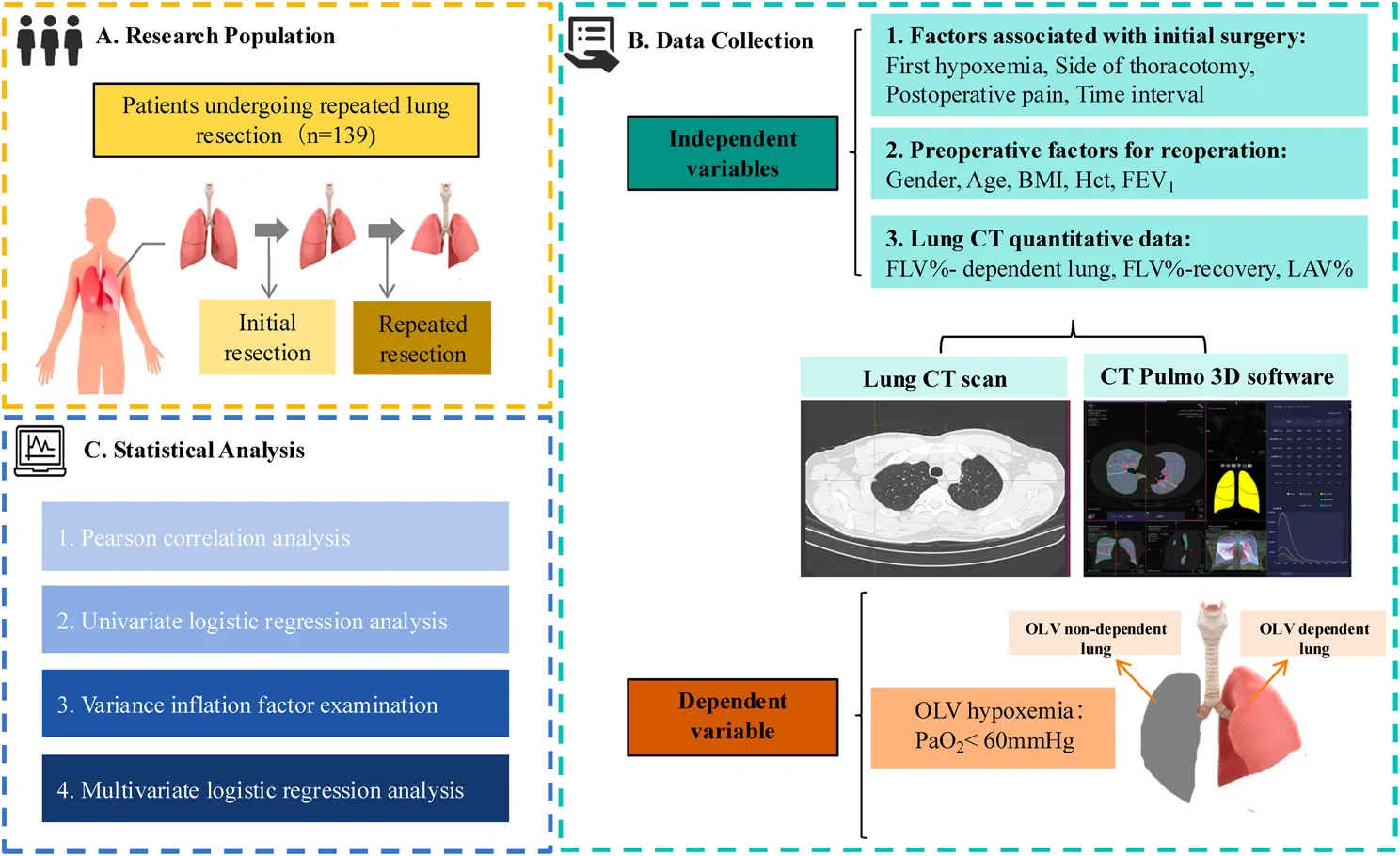
Schematic diagram of this study design

This section describes the research design of this study, which is schematized in Fig. 1. It consists of three components: research population, data collection, and statistical analysis.

### Research population

This was a retrospective study approved by the Institutional Review Board (IRB) of Yunnan Cancer Hospital (approval No. KYLX2023-191). The study protocol adhered to the ethical principles outlined in the Declaration of Helsinki (2013 revision). Given the retrospective design and exclusive use of anonymized data, the ethics committee waived the requirement for written informed consent. We collected clinical data on patients who underwent repeated lung resection at Yunnan Cancer Hospital between January 2022 and December 2023. The inclusion criteria for the study were as follows: (1) Patient age ≥ 18 years; (2) American Society of Anesthesiologists (ASA) physical status classification of I-II. Since in our center, ASA III–IV patients rarely undergo repeated lung resection, and when they do, management is often individualized rather than based on standard OLV pathways; (3) The OLV tool employed was the DLTs. (4) The primary surgical approach was thoracoscopic lobectomy with systematic lymph node dissection. The following patients were excluded: those with incomplete data (e.g., information related to the initial surgery could not be obtained or preoperative CT images could not be quantitatively analyzed), and those with specific preoperative or intraoperative conditions that could lead to hypoxemia (e.g., intraoperative hemorrhagic shock or severe allergies, etc. preoperative lung infection or preoperative peripheral pulse oxygen saturation (SPO_2_) <90%). A total of 139 patients met the inclusion criteria and were ultimately included in the study.

### Data collection

Patient information was obtained from the hospital medical record system. Variables were selected based on prior studies [7, 9–13] and clinical practice. Data from the following three sections were included as study independent variables: important data related to the initial surgical procedure, preoperative factors for reoperation, and lung CT quantitative data, while intraoperative variables were not incorporated given the study’s focus on preoperative risk stratification. The occurrence of hypoxemia during OLV as an outcome indicator was included as a dependent variable, as detailed in Table 1.

**Table 1:** List of data included in this study

Factors associated with initial surgery	Preoperative reoperation factors	Quantitative CT-derived variables	Dependent variable
First hypoxemia(yes/no)	Gender (male/female)	FLV%- dependent lung	OLV hypoxemia
Side of thoracotomy (ipsilateral/contralateral)	Age (years)	FLV%-recovery	
Postoperative pain (yes/no)	BMI (kg/m^2^)	LAV%	
Time interval (months)	Hct (%)		
	FEV_1_ (% predicted)		
	DLCO (% predicted)		

Abbreviations: BMI, body mass index, CT, computed tomography, DLCO, diffusing capacity for carbon dioxide, FEV_1_, forced expiratory volume in 1 second, FLV, functional lung volume, Hct, hematocrit, LAV, low attenuating volume, OLV, one-lung ventilation

#### Important data related to the initial surgical procedure

The impact of a primary lung resection on the incidence of hypoxemia in a subsequent lung resection remains uncertain. Therefore, we collected data regarding the initial procedure. This included whether hypoxemia occurred during the initial lung resection (first hypoxemia), whether the open side of the reoperation was ipsilateral or contralateral compared with the initial operation, whether moderate-to-severe pain occurred at 48 hours postoperatively (postoperative pain), and the time interval between the two operations (in months). The level of pain was determined on the basis of the visual analog scale (VAS) score recorded by the anesthesia nursing staff at the 48-h postoperative follow-up visit, and VAS ≥ 4 was considered to have experienced moderate to severe pain [19].

#### Preoperative factors for reoperation

Based on a review of the existing literature [20–22], we identified several key preoperative indicators as study variables. These included: patients’ age, gender, BMI, smoking history, serum Hct, forced expiratory volume in 1 second (FEV_1_) as a percentage of predicted value[FEV_1_ (% predicted)], and DLCO as a percentage of predicted value[DLCO (% predicted)]. Lung function indices were obtained using the MasterScreen Body examination (CareFusion Germany 234 GmbH, Hochberg, Germany), and the results were averaged after the patients underwent two examinations. Smoking history was defined as either still smoking prior to surgery or having a history of smoking lasting more than 1 y. Conversely, never smoking or a smoking history of less than 1 year was considered to be no smoking history [27].

#### Lung CT quantitative data

The determination of quantitative lung CT data was achieved through a three-step process: chest CT scanning, CT image reconstruction, and image post-processing analysis. All patients underwent whole lung scanning in the supine position during deep inspiration and breath-hold, with the following scanning parameters: tube current 110 mAs, collimation 0.6 mm, rotation time 0.5 s, and pitch 1.2. The acquired CT images were then reconstructed into cross-axial slices with a thickness of 1 mm using an iterative reconstruction algorithm. Finally, the reconstructed CT image data were uploaded to the syngo.via workstation (VB20A, Dual Energy, Siemens Healthineers) and analyzed using CT Pulmo 3D software (syngo.via, Siemens Healthineers, Germany) for quantitative data analysis. The software employs a threshold segmentation method to automatically segment the lung structures and generate a 3D model. Subsequently, it automatically calculates the lung attenuation values and determines the FLV% and LAV% based on different ranges of attenuation values. Attenuation coefficients are quantified in Hounsfield units (HU), providing a surrogate metric for pulmonary tissue density. In the field of radiology, FLV is operationalized as the pulmonary volume exhibiting attenuation values ranging from −600 to −910 HU [23]. LAV refers to the lung parenchymal volume exhibiting attenuation values lower than −950 HU [24]. The study aimed to quantify three pulmonary CT metrics: the proportion of FLV in the dependent lung relative to total lung FLV (FLV%-dependent lung), the proportion of total lung FLV before reoperation relative to that before the initial operation (FLV%-recovery), and LAV%.

The CT Pulmo 3D software’s cross-scanner compatibility has been validated in multicenter studies [25], and its algorithms are analogous to other validated platforms with high inter-software concordance [18, 26, 28–30]. Automated outputs were reviewed by a senior radiologist blinded to clinical outcomes, with manual correction performed only when segmentation quality was deemed inadequate.

#### Intraoperative anesthetic management

Standard intravenous access and peripheral arterial catheterization for invasive blood pressure monitoring were established prior to surgery. Intraoperative monitoring included electrocardiogram, invasive blood pressure, SpO₂, and PETCO₂. Following intravenous anesthesia induction, a DLT (Shiley™ endobronchial tube accessories; Covidien, Mansfield, US) was inserted. The DLT was selected contralateral to the thoracotomy side: a left DLT for right thoracotomy and a right DLT for left thoracotomy. An appropriately sized DLT was selected based on the anteroposterior tracheal diameter measured on chest X-ray or CT. Correct placement was confirmed by chest auscultation and fiberoptic bronchoscopy in the supine position, and reconfirmed in the lateral position. During OLV, the tidal volume was set to 5–6 ml/kg IBW, the respiratory rate was 8–12/min, the FiO_2_ was 100%, the partial pressure of carbon dioxide in arterial blood (PaCO_2_) was maintained in the normal range, and the positive end-expiratory pressure (PEEP) was set to 5–10 cmH_2_O when necessary. Fluid management followed a restrictive strategy.

#### Outcome indicator

The outcome indicator for this study was the occurrence of hypoxemia during OLV. Arterial blood was drawn during the operation for blood gas analysis (Cobas b 221, Roche Diagnostic, India), from which PaO_2_ was obtained. Hypoxemia during OLV was defined as PaO_2_ < 60 mmHg after a 30-minute OLV period [31, 32]. Following the onset of hypoxemia, the anesthesiologist took necessary steps to ensure the patient’s oxygenation status was within the safe range, including precise DLT placement, recruitment maneuver, uninterrupted positive-pressure application to the operative lung, and low-tidal-volume dual-lung ventilation.

### Validation metrics

To enhance transparency regarding the behavior of the multivariable model in this small sample, we performed 1000-iteration bootstrap resampling with replacement. This procedure was intended to provide descriptive insight into model discrimination and calibration, not to validate a clinical prediction tool. The area under the receiver operating characteristic (ROC) curve (AUC) with its 95% confidence interval, sensitivity, and specificity were calculated. Calibration was assessed using the Hosmer-Lemeshow goodness-of-fit test and a calibration curve.

### Statistical analysis

All statistical analyses were conducted using the IBM SPSS 27.0 software. Model performance assessment was conducted in R (version 4.5.1) and Python (version 3.10) software environments. Continuous variables were summarized as mean ± standard deviation (SD). Categorical variables including: gender, smoking history, first hypoxemia, postoperative pain, and side of thoracotomy were coded as 0 and 1. Missing data were assessed before analysis. No missing values were present in the final analytic dataset; therefore, imputation was not required.

The analytical process consisted of several sequential steps: First, Pearson correlation analysis was conducted to evaluate the linear relationships among the variables. Second, OLV hypoxemia served as the outcome variable, and univariate logistic regression was performed to screen for candidate risk factors. Third, variables that showed statistical significance (*p* < 0.05) in univariate logistic regression were subjected to a variance inflation factor (VIF) examination to ascertain the absence of multicollinearity among the variables. Finally, these risk factors were included in multivariate logistic regression analysis following confirmation of no linear interference between variables. The final multivariable model included 5 variables based on 23 events (events per variable =  4.60), a ratio supported by Vittinghoff and McCulloch’s simulation study for factor-identification studies with careful variable control [33]. A *p*-value threshold of <0.05 was adopted for statistical significance across all analyses.

## Results

### Results of descriptive statistics and correlation analysis of preoperative variables in 139 patients

Of the 142 patients initially included in the study, two were excluded due to incomplete information, and one was excluded due to the absence of intraoperative blood gas results. Ultimately, a total of 139 patients who had undergone repeated lung resection were included in the study, of whom 23 (16.55%) developed intraoperative hypoxemia. Of the 139 patients, 46 (39.66%) were male, with a mean age of 55.98 years and a mean BMI of 22.99 kg/m^2^. Descriptive statistics of the preoperative variables are detailed in Table 2.

**Table 2:** Descriptive statistical analysis results of research variables

Characteristics	Mean ± SD
Gender	Male:54(38.85%); female:85(61.15%)
Age(years)	55.98 ± 8.43
BMI (kg/m^2^)	22.99 ± 2.68
Smoking history, n (%)	Yes:20(14.30); no:119(85.60)
Hct (%)	42.99 ± 3.86
FEV_1_ (% predicted)	92.49 ± 15.71
DLCO (% predicted)	105.27 ± 21.23
First hypoxemia, n (%)	Yes:9(6.47%); no:130(93.53)
Side of thoracotomy, n (%)	contralateral: 120(86.33); ipsilateral:19(13.67)
Postoperative pain, n (%)	Yes:34 (24.46); no:105(75.54)
Time interval (months)	25.06 ± 30.58
FLV%-dependent lung	46.35 ± 7.55
FLV%-recovery	90.42 ± 11.55
LAV%	30.90 ± 11.79

Abbreviations: BMI, body mass index; DLCO, diffusing capacity for carbon dioxide; FEV_1_, forced exhalation volume in 1 s; FLV, functional lung volume; Hct, hematocrit; LAV, low attenuation volume; SD, standard deviation

**Fig. 2:**
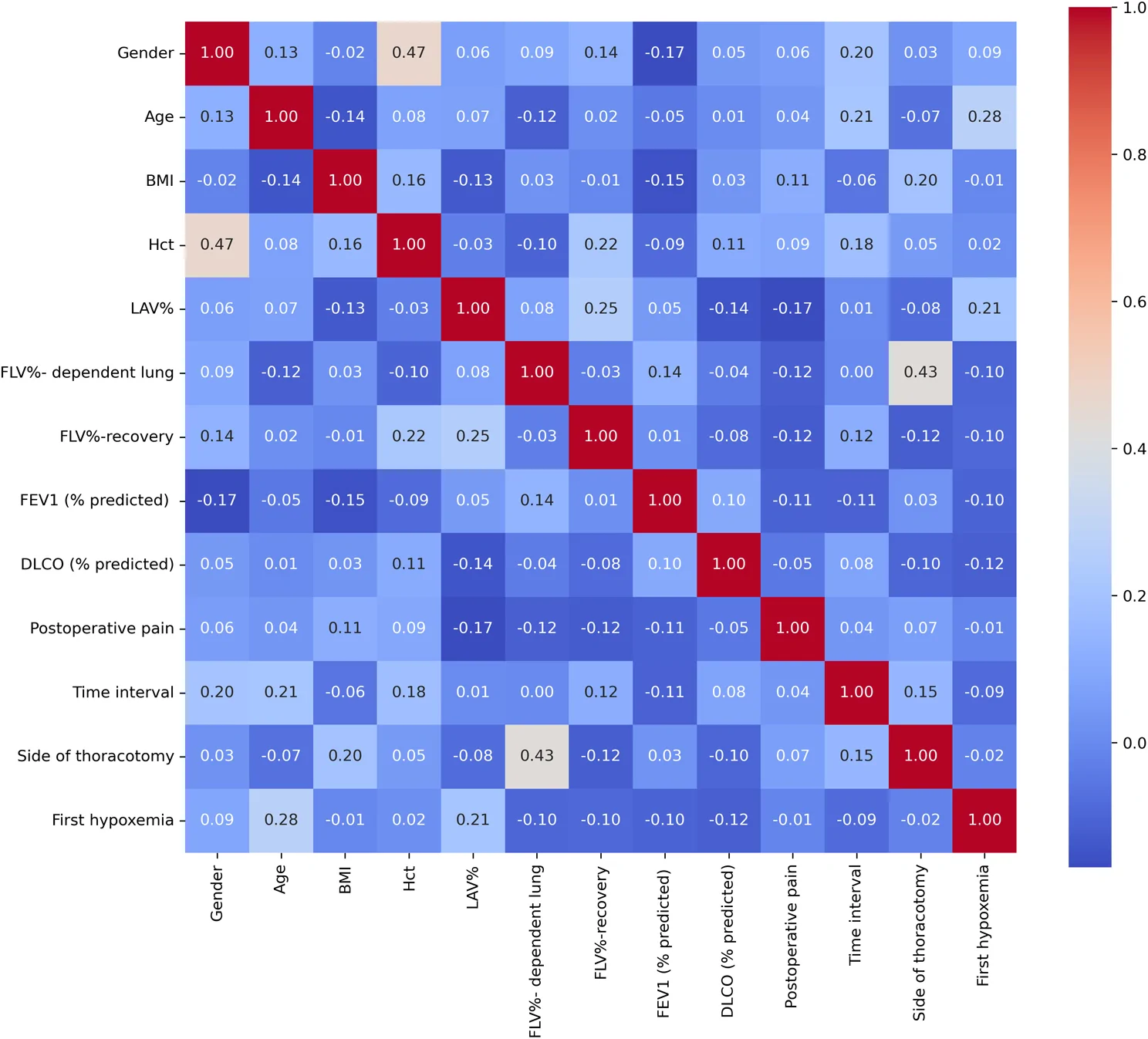
Correlation matrix of independent variables

Pearson correlation analysis revealed no strong linear relationships among the independent variables (all |r| <0.5; Fig. 2), confirming their suitability for simultaneous inclusion in the multivariable model.

### Results of univariate logistic regression screening for preoperative risk factors

Results of the univariate logistic regression analysis, with OLV hypoxemia as the dependent variable, are shown in Table 3. Analysis identified five preoperative variables that demonstrated significant associations with OLV hypoxemia(*p* < 0.05). These were: FEV_1_(% predicted) (*p* = 0.013), first hypoxemia (*p* = 0.031), postoperative pain (*p* = 0.006), FLV%-dependent lung (*p* < 0.001), and FLV%-recovery (*p* < 0.001).

**Table 3:** Results of univariate logistic regression analysis of preoperative risk factors for OLV hypoxemia

Characteristics	Coefficient	*p* Value
Gender	0.209	0.662
Age (years)	0.049	0.092
BMI (kg/m^2^)	0.066	0.427
Smoking history	0.135	0.841
Hct (%)	0.023	0.705
FEV_1_ (% predicted)	−0.040	0.013^*^
DLCO (% predicted)	−0.008	0.492
First hypoxemia	−1.542	0.031^*^
Side of thoracotomy	0.589	0.453
Postoperative pain	−1.310	0.006^*^
Time interval (months)	−0.002	0.757
FLV%-dependent lung	−0.265	<0.001^*^
FLV%-recovery	−0.181	<0.001^*^
LAV%	−0.032	0.126

Abbreviations: BMI, body mass index; CI, confidence interval; DLCO, diffusing capacity for carbon dioxide; FEV_1_, forced exhalation volume in 1 s; FLV, functional lung volume; Hct, hematocrit; LAV, low attenuation volume; OR, odds ratio

*The asterisk indicates that the variable was statistically significant

### Results of VIF test and multivariate logistic regression analysis of risk factors influencing hypoxemia

Following univariate filtering (*p* < 0.05), five risk factors entered the VIF diagnostics. It is usually assumed that when 0 < VIF <5, there is no multicollinearity. The results showed no linear correlation between the variables (Mean VIF = 1.036, Min VIF = 1.029, Max VIF = 1.043), as detailed in Table 4.

**Table 4:** Results of multicollinearity analysis of independent variables

Variables	VIF
FEV_1_(% predicted)	1.038
First hypoxemia	1.030
Postoperative pain	1.042
FLV%-dependent lung	1.043
FLV%-recovery	1.029

Abbreviations: FEV_1_, forced exhalation volume in 1 s; FLV, functional lung volume; VIF, variance inflation factor

A multivariable logistic regression model was constructed to identify independent risk factors for OLV hypoxemia, incorporating five candidate risk factors. The results showed that FLV%-dependent lung was a significant protective factor (odds ratio [OR] 0.92, 95% confidence interval [CI] 0.85–0.99; *p* = 0.034). Conversely, first hypoxemia was a strong risk factor (OR 7.64, 95%CI 1.65–35.31; *p* = 0.009), indicating nearly 7.6-fold increased odds of hypoxemia. Postoperative pain was also significantly associated with increased risk (OR 5.59, 95%CI 1.95–16.03; *p* = 0.001), while FEV_1_% predicted showed a modest effect (OR 1.04, 95%CI 1.00–1.08; *p* = 0.038). Detailed effect estimates for all risk factors are provided in Table 5. Clinically, each 10% increase in FLV%-dependent lung corresponds to an approximately 56% relative risk reduction for OLV hypoxemia. The large odds ratios observed for prior hypoxemia (7.64) and postoperative pain (5.59) indicate that these history-based factors may serve as high-yield preoperative alert flags, whereas the modest per-unit effect of FEV_1_% predicted (OR 1.04) suggests limited clinical impact across typical observed ranges.

**Table 5:** Results of multivariate logistic regression analysis of preoperative risk factors influencing hypoxemia during OLV

Characteristics	Coefficient	OR (95% CI)	*p* Value
FEV_1_(% predicted)	0.037	1.04 (1.00–1.08)	0.038^*^
First hypoxemia	2.033	7.64 (1.65–35.31)	0.009^*^
Postoperative pain	1.721	5.59 (1.95–16.03)	0.001^*^
FLV%-dependent lung	−0.084	0.92 (0.85–0.99)	0.034^*^
FLV%-recovery	−0.026	0.98 (0.94–1.01)	0.164

Abbreviations: CI, confidence interval; FEV_1_, forced exhalation volume in 1 s; FLV, functional lung volume; OR, odds ratio

*The asterisk indicates that the variable was statistically significant

### Exploratory model performance

**Fig. 3:**
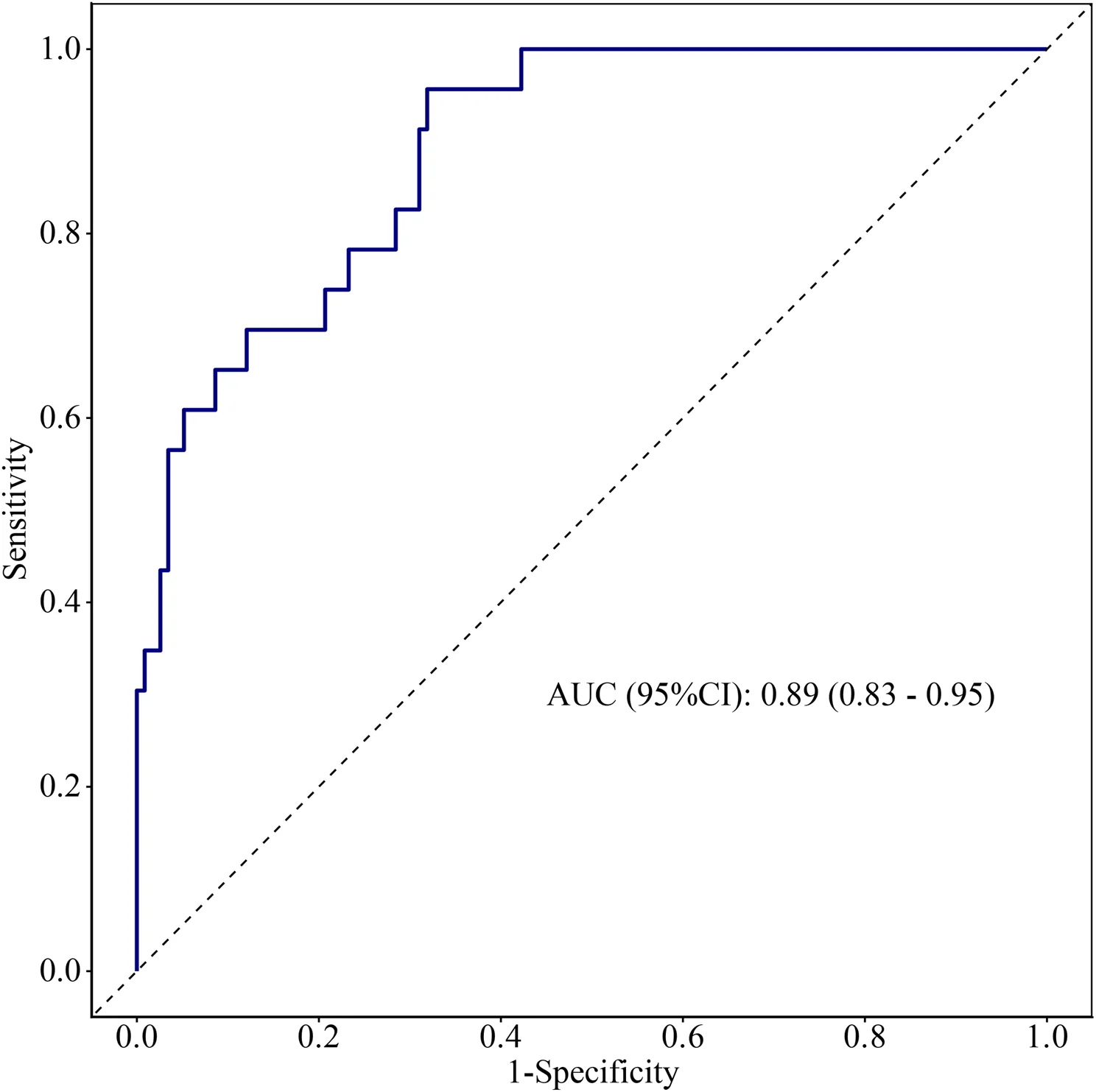
ROC curves of multivariable model. AUC, area under the curve; CI, confidence interval

**Fig. 4:**
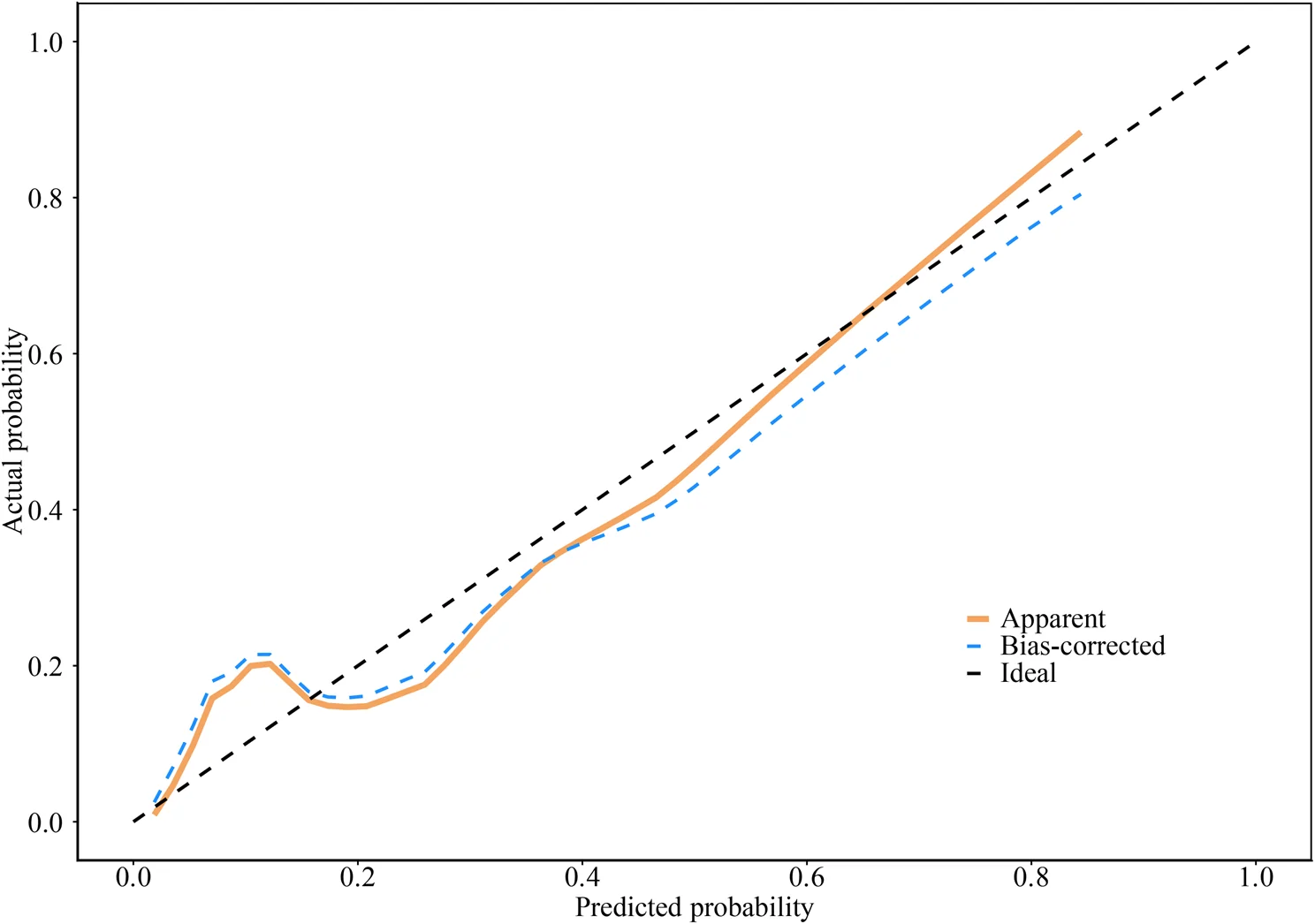
Calibration curve

The multivariable model demonstrated an AUC of 0.89 (95% CI: 0.83–0.95) (Fig. 3).Sensitivity was 96% (95% CI: 0.87–1.00), specificity 68% (95% CI: 0.60–0.77). The Hosmer-Lemeshow test indicated statistically adequate calibration (χ^2^ = 3.86, *p* = 0.870) (see Table 6). However, the bootstrap bias-corrected calibration curve revealed systematic overestimation of predicted probabilities compared with the ideal calibration line (Fig. 4), consistent with overfitting in this small sample. These metrics are provided for descriptive transparency only and should not be interpreted as validating a clinical prediction model.

**Table 6:** Bootstrap-derived performance metrics of the multivariable logistic regression model

Validation metrics	Value (95% CI)
AUC	0.89 (0.83–0.95)
Accuracy	0.73 (0.64–0.80)
Sensitivity	0.96 (0.87 - 1.00)
Specificity	0.68 (0.60 - 0.77)
Hosmer-Lemeshow test	χ^2^ = 3.86, *p* = 0.870

Abbreviations: CI, confidence interval; AUC, Area under the curve

### Multivariate logistic regression Results comparing OLV hypoxemia between repeated and initial pneumonectomy procedures

**Fig. 5:**
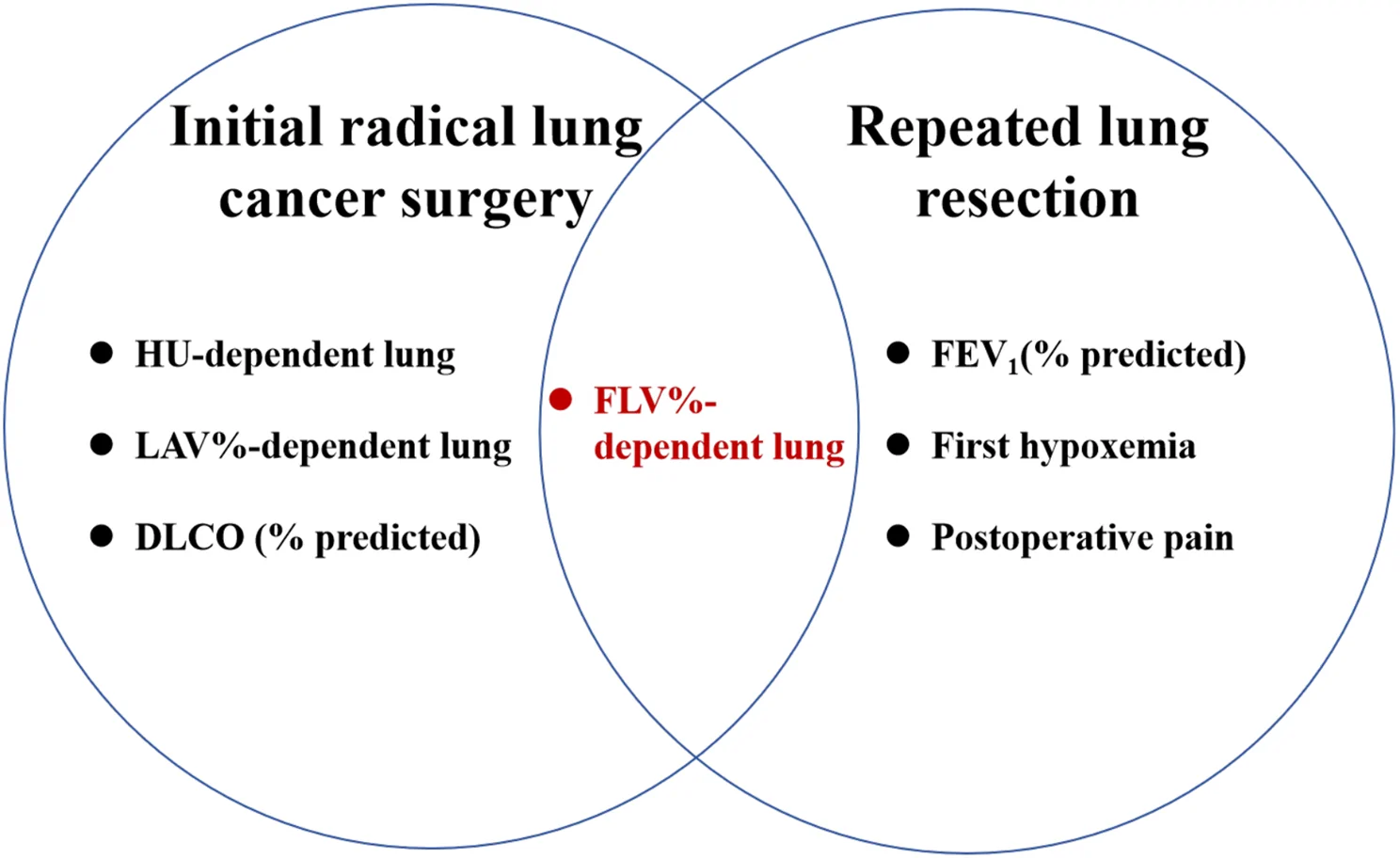
Multivariate logistic regression analysis of hypoxemia risk: initial radical lung cancer surgery versus repeated lung resection. DLCO, diffusing capacity for carbon dioxide, FEV_1_, forced expiratory volume in 1 second, FLV, functional lung volume, LAV, low attenuating volume, OLV, one-lung ventilation

Our previously published findings indicated that three quantitative pulmonary CT parameters—FLV%, HU, and LAV% in OLV-dependent lungs—along with one pulmonary function parameter—DLCO (% predicted)—were independently linked to the risk of hypoxemic events in patients undergoing primary pulmonary oncological resection [27]. In contrast, the findings of this study differ. Only one quantitative CT parameter retained independent significance for hypoxemia risk throughout repeated lung resection: the FLV% in OLV-dependent lungs. Regarding pulmonary function indicators, the independent risk factor was FEV_1_ (% predicted) rather than DLCO (% predicted). The other two independent risk factors are indicators closely associated with the initial surgery—First hypoxemia and Postoperative pain, as shown in Fig. 5.

## Discussion

Hypoxemia associated with OLV in patients undergoing repeated lung resection remains a critical consideration for anesthesiologists. This condition is pivotal in dictating the selection of lung isolation instruments preoperatively and in shaping the strategic decisions regarding respiratory management during OLV. In this study, the incidence of OLV hypoxemia was found to be 16.55% in patients who underwent repeated lung resection. The main findings of this study were FLV%-dependent lung (OR 0.92, 95% CI 0.85–0.99; *p* = 0.034), first hypoxemia (OR 7.64, 95% CI 1.65–35.31; *p* = 0.009), postoperative pain (OR 5.59, 95% CI 1.95–16.03; *p* = 0.001), and FEV_1_(% predicted) (OR 1.04, 95% CI 1.00–1.08; *p* = 0.038) were significantly associated with the development of hypoxemia during OLV in repeated lung resection.

### Higher dependent-lung FLV% is associated with lower OLV hypoxemia risk in repeated lung resection

Greater dependent-lung FLV% was associated with lower odds of hypoxemia (OR 0.92, 95% CI: 0.85–0.99, *p* = 0.034), suggesting a protective effect of functional lung volume in the ventilated lung. Ueda et al. [14] reported that improvements in postoperative pulmonary function following lobectomy and segmental resection were proportional to the increase in FLV. This reflects that FLV (−600 to −910 HU) represents true functional gas-exchanging tissue, excluding non-functional areas such as emphysema or fibrosis. Fan et al. [7] quantified the proportion of total FLV encompassed by the planned resection for postoperative spirometric projection with improved accuracy, underscoring that functional tissue distribution determines respiratory outcomes.

Mechanistically, during lateral one-lung ventilation, the dependent lung remains ventilated while the non-dependent (operative) lung is collapsed. Gravity directs increased blood flow to the dependent ventilated lung; reduced FLV% in this region contributes to ventilation-perfusion mismatch and impaired oxygenation [20]. Additionally, post-resection compensatory expansion is heterogeneous; certain regions (e.g., middle lobe) demonstrate greater functional reserves through enhanced diaphragmatic contact [9]. Our data indicate that a lower dependent-lung FLV%—and thus its reduced functional share—is significantly associated with increased probability of OLV hypoxemia. This parameter, readily extracted from conventional CT data, may aid preoperative risk evaluation.

### Comparison of differences in lung CT quantitative indicators as risk factors for intraoperative hypoxemia between primary and repeated lung resections

Preoperative HU and LAV% are significant risk factors for intraoperative hypoxemia in primary lung resection [14, 34, 35], but were not associated with hypoxemia in our repeated resection cohort. This divergence may reflect a fundamental pathophysiological shift: in primary resection, HU and LAV% are thought to reflect baseline parenchymal destruction (emphysema severity and tissue density loss), which may limit oxygenation reserve. Following initial lobectomy, however, compensatory expansion and decreased density in the residual lung may represent physiological adaptation rather than progressive pathology [7]; the LAV% signal becomes obscured by post-surgical remodeling, potentially rendering baseline emphysema metrics non-predictive in the reoperative setting.

Consequently, FLV%-dependent lung emerges as the sole CT predictor retained across both surgical contexts. In primary resection, FLV% reflects functional tissue available for ventilation; in repeated resection, it captures post-compensatory functional redistribution—specifically, whether the dependent lung has achieved sufficient volume recovery to sustain oxygenation during OLV. This parameter thus indexes local pulmonary function redistribution rather than baseline parenchymal quality, explaining why HU and LAV% lose predictive value while FLV%-dependent lung remains critical. This contrast implies that risk stratification strategies developed for primary lung resection cannot be directly extrapolated to repeated procedures, where functional volume assessment takes precedence over baseline parenchymal characterization.

### First hypoxemia and postoperative pain were significantly and positively correlated with hypoxemia during OLV in repeated lung resection

The second major finding reveals that two factors associated with initial lung resection are significantly associated with hypoxemia during repeated lung resection [5, 36]. First hypoxemia was associated with a 7.64-fold increased odds (95% CI: 1.65–35.31, *p* = 0.009), aligning with the 6.49% incidence reported in prior studies [36], attributed to reduced ventilation/perfusion ratio and increased intrapulmonary shunt [5]. Anesthetists should be aware of this association when evaluating patients for redo surgery. The second factor, postoperative pain, was also significantly associated with hypoxemia during OLV. Moderate-to-severe acute pain may evolve into chronic pain [37, 38], reduces residual lung expansion [39], and is strongly associated with poor lung function recovery [40]. This suggests that pain management during initial surgery may warrant attention in preoperative assessment for repeated lung surgery, though prospective studies are needed to determine whether optimizing pain control could modify hypoxemia risk.

### FEV_1_(% predicted) was significantly and positively associated with hypoxemia during OLV in repeated lung resection

Our results demonstrated a statistically significant positive association between preoperative FEV₁ (% predicted) and intraoperative hypoxemia during one-lung ventilation in repeat lung resection, consistent with prior observations in first-time pulmonary surgery [41, 42]. However, the underlying mechanisms may differ. In initial resections, this paradoxical phenomenon has been hypothesized to result from slower collapse of the non-ventilated lung and auto-PEEP effects in patients with obstructive physiology [42]. For repeated resections, we speculate that patients with lower FEV_1_%—reflecting greater prior parenchymal loss—may have differential pulmonary vascular responses to OLV compared with those with preserved FEV_1_%, though this hypothesis lacks direct empirical support and should be interpreted cautiously. Regardless of mechanism, the modest magnitude of this association (OR 1.04 per 1% increase) suggests limited clinical relevance across the typical observed range.

### Limitations of the study and perspectives for the future

This study has several limitations. First, its single-center retrospective design with a limited sample size may constrain the generalizability of findings. We acknowledge that this events-per-variable ratio is modest and that findings should be interpreted cautiously. Although we performed 1000-iteration bootstrap resampling to provide exploratory insight into model behavior, the resulting metrics derive entirely from internal resampling of the derivation cohort and do not constitute model validation. External validation would be needed in future multicenter studies. Second, the cohort was restricted to ASA physical status I-II patients. This restriction reflects actual clinical practice in our center, but it precludes direct application of our findings to higher-risk populations. Third, we defined hypoxemia as PaO₂<60 mmHg at a single time point, which represents a simplified binary outcome that may not fully capture the dynamic and continuous nature of intraoperative oxygenation changes. Fourth, formal inter-observer or intra-observer reliability testing for CT-derived quantitative measurements was not performed. While automated segmentation with standardized HU thresholds reduces operator-dependent variability, and all outputs were reviewed by a board-certified radiologist, the absence of quantitative reproducibility metrics limits assessment of measurement robustness. Fifth, although our institutional perioperative protocol standardized anesthetic management, unmeasured intraoperative variability may have influenced oxygenation and represents a potential source of residual confounding. Finally, we employed a *p*-value threshold of <0.05 for variable selection in the multivariate model. While this conservative approach reduces false-positive risk in our small sample, it may have excluded variables with modest but real effects. Future research will establish a prospective observational cohort with standardized perioperative data collection, enabling development of a validated clinical prediction tool for intraoperative hypoxemia. Additionally, future studies could employ and compare machine learning approaches (e.g., LASSO, decision tree) to develop robust prediction tools, though these were not feasible in the current study given sample constraints. Furthermore, formal inter-observer and intra-observer reliability assessment, including ICC calculation and Bland-Altman analysis, will be incorporated to validate the reproducibility of CT-derived quantitative biomarkers.

## Conclusion

This study identified four preoperative factors independently associated with intraoperative hypoxemia during repeat lung resection: FLV%-dependent lung (CT-derived quantitative biomarker), first hypoxemia and postoperative pain (initial surgery-related indicators), and FEV_1_% predicted (pulmonary function indicator). These findings require validation in larger, multicenter prospective cohorts before informing clinical practice. If confirmed, they suggest that anesthesiologists should pay close attention to the compensatory capacity of dependent lung, as reflected by the FLV%-dependent lung and FEV_1_ (% predicted), following initial lung resection. Similarly, attention to prior intraoperative hypoxemia and acute postoperative pain control during initial surgery may warrant consideration in preoperative evaluation for redo procedures. Until such validation is available, these results should be considered hypothesis-generating rather than practice-changing.

## Data Availability

The datasets used and/or analysed during the current study are available from the corresponding author on reasonable request.
